# Macrophage: SHIP of Immunity

**DOI:** 10.3389/fimmu.2014.00620

**Published:** 2014-12-04

**Authors:** Charles D. Mills, Anita C. Thomas, Laurel L. Lenz, Markus Munder

**Affiliations:** ^1^BioMedical Consultants, Marine on St. Croix, MN, USA; ^2^Bristol Heart Institute and Bristol CardioVascular, University of Bristol, Bristol, UK; ^3^Immunology and Microbiology Department, University of Colorado School of Medicine, Aurora, CO, USA; ^4^Third Department of Medicine, University Medical Center, Johannes Gutenberg University, Mainz, Germany

**Keywords:** macrophage, M1, M2, nitric oxide, iNOS, arginase, wound, cancer

Immunology. Why does it exist? Two words. Cure disease. People get diseases. “Test tubes” do not. People fund immunologists for solutions to their health problems. But, immunologists often study leukocytes in test tubes – the laboratory – away from diseases. Why? Because much can be learned from analyzing cellular biochemistry and behaviors *in vitro* that cannot be ascertained when leukocytes are in animals. At the same time, isolated leukocyte reactions often do not reflect how the immune system operates as a unit. So, it is critical to verify *in vitro* observations *in vivo*. Among leukocytes, macrophages are the central initiating and directing element in immune systems, and serve this role through four basic “SHIP” functions *in vivo*: Sample; Heal; Inhibit; and Present (antigen) (1–4). The polar-opposite functions of Heal (M2-type) and Inhibit (M1-type) can have profoundly different effects on host survival, and require unique and major changes in macrophage metabolism and physiology. In turn, macrophage populations are necessarily heterogeneous as they adapt to protect hosts in different ways: they exhibit “plasticity.” Some have focused on measuring ever-expanding lists of cell surface or various other “markers” (mostly *in vitro*) to try and sub-type macrophages. But, the “heterogeneity” created by such studies can be “illusory” because there are many more markers than there are functions (e.g., M1/inhibit and M2/heal). Thus, it is important to focus on classifying macrophages by functions, such as SHIP, to navigate through a “sea of plasticity.” And, thereby realize the enormous potential of macrophages/innate immunity for improving health.

## Basic Macrophage Functions *In vivo*

The earliest *in vivo* SHIP function observed in macrophages was their ability to “sample” by ingesting items in their surroundings (5, 6). Through sampling, macrophages routinely receive “self” signals that instruct them to repair or replace lost or effete cells and intercellular matrices. The heal-type function of macrophages is now called M2 [(7), reviewed in Ref. (2)]. Following infection (or trauma), M2/heal-type macrophages can rapidly switch to become M1/inhibit-type, to promote host defense (1). M2/heal responses are mediated by ornithine, and other growth-promoting molecules (8, 9). M1/inhibit is mediated by nitric oxide (NO) and other molecules that promote cellular killing activity (10, 11). Fascinatingly, both ornithine and NO arise from one amino acid: arginine (12).

**Figure 1:**
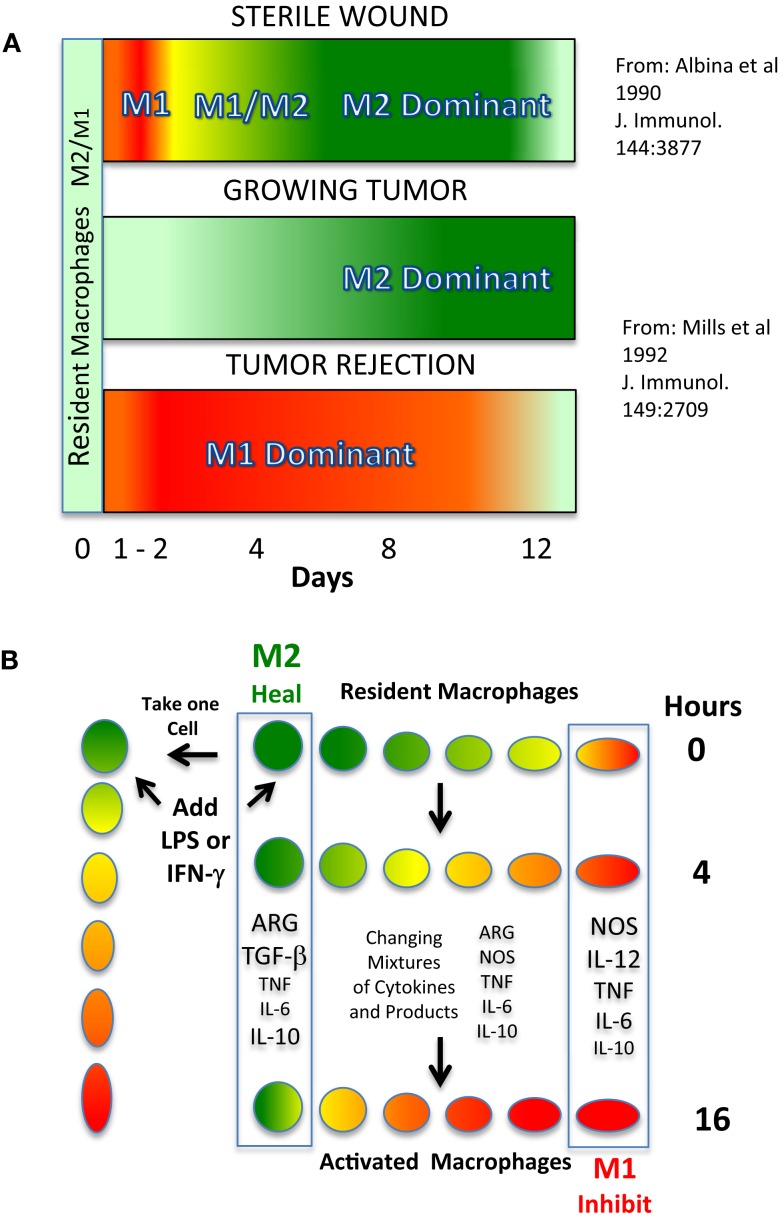
**(A)**. Predominance of M1 or M2-dominant macrophage responses *in vivo* in different types of inflammation. Top: a wound results in a strong, but brief, shift from local macrophages making primarily ornithine to making NO. Wound macrophages then become M2-dominant to aid in wound healing. Middle: a growing tumor elicits macrophages, and macrophage production of NO is suppressed and ornithine production predominates. Bottom: if a host is vaccinated against a tumor, implantation of the tumor results in T cell-dependent M1 dominance and the tumor is rejected. **(B)** M2 to M1-type macrophage conversion and the illusion of “subsets.” Top: resident macrophages primarily produce ornithine via arginase (Arg). Middle: upon stimulation, macrophage production of iNOS/NO increases. And the production of other cytokines, etc., changes during this time. Bottom: after 16 h, macrophage NO production increases further and ornithine production declines.

The biochemical basis for the M2/heal function of macrophages was discovered before the M1/inhibit function (8, 12). As illustrated in Figure 1A (top), in sterile wounds, macrophages produce ornithine (a precursor of polyamines and collagen for repair) as healing proceeds (Green – M2 dominance) (13). Around the same time, it was observed that macrophages in growing tumors were also the M2/ornithine-producing type (Figure 1A middle). This latter finding provided a biological explanation for the association of intratumor macrophages with tumor growth (14). M2/heal-type macrophages have since been shown to also dominate in human tumors, and are associated with poor survival (15–18).

The biochemical basis of how M1/inhibit-type macrophages kill pathogens (or, abnormal “self”) also came from the study of wounds and cancer described above, as well as other studies [(19–26), reviewed in Ref. (3, 12)]. As mentioned, macrophages have a unique ability to switch from making the heal molecule, ornithine, *in vivo* to making the killer molecule, NO (1). Such a switch is shown in Figure 1A (top). For a brief period following wounding, a high concentration of NO is present (M1 activity, Red), which can protect the wound if infectious agents have been introduced (13). It is not clear exactly what stimuli cause this injury-induced NO production, though neutrophils are also involved (27, 28). If the wound is sterile, macrophage NO production stops, neutrophil emigration ends, and wound macrophages make ornithine (M2 activity) as mentioned. Another example of the key protective function of macrophages making NO is shown in Figure 1A (bottom). It can be seen that if a host is vaccinated against the tumor shown in Figure 1A (middle), implantation of the tumor causes intratumor macrophages to make a large quantity of NO that helps cause tumor rejection (12). Macrophage NO is also an important defense against a variety of infectious diseases (29). That M2/ornithine or M1/NO are important effector molecules are supported by studies showing that interference with these activities *in vivo* alters healing or host protection [reviewed in Ref. (12)]. Conversely, overexpression of M1/inhibit responses is associated with conditions such as atherosclerosis and arthritis, while M2-type contribute to chronic infections, promotion of tumor growth, and allergies (3, 29). Together, these results demonstrated two core functions that result from macrophages sampling their environment, and that affect health in very important, and opposite, ways: the M1/inhibit response and the M2/heal response.

Inhibit-type macrophages and heal-type macrophages were specifically renamed M1 and M2 because these macrophage responses [or dendritic cells^1^ (30–32)] were also found to stimulate T cells to make Th1-type (cellular-based), or Th2-type (antibody-based) cytokines (7), respectively. This fourth SHIP function of present (antigen) is only expressed in vertebrates (1). Although T cells can produce molecules that “activate” or “alternatively activate” macrophages (33, 34), macrophages evolved first and respond first. They directly sense Pathogen or Damage – Associated Molecular Patterns (PAMP or DAMP) that can initiate M1 or M2-type responses (35–39). Subsequently, macrophages can stimulate T cells (that cannot recognize antigens directly), and thereby further amplify M1 or M2 responses. This “secondary” type of T cell-driven response (macrophage “activation”) was discovered by Mackaness and colleagues using mice preimmunized to *Listeria* and other pathogens (40). It was not known at this time that macrophages were actually responsible for initially activating T cells (41, 42). The recent revelation about the central role of macrophages in immune responses caused a sea change in understanding how immune responses occur and are regulated *in vivo* (1, 7).

## Biochemical and Physiologic Host Elements that Influence How Macrophages Protect Hosts

Macrophage SHIP functions (sample, heal, inhibit, and present) are regulated by integration of a variety of endogenous (e.g., host-derived and resident microbiota) and exogenous signals (1, 43). For example, in the absence of infection or injury, TGF-β helps maintain macrophages in the routine M2/heal mode (7). Other host-derived molecules, such as oxidized LDL, can stimulate M1-type responses that contribute to atherosclerosis (4, 44). Following infection or injury, certain PAMPs and DAMPs stimulate macrophages to switch from M2/heal to M1/inhibit mode (35–37). IFN-γ was shown to be the primary T cell product that further amplifies M1/inhibit activity (45). Later, macrophage IL-12 was found to be a key cytokine (along with increased Class II MHC expression) that stimulates IFN-γ production by T cells (41, 42, 46). Macrophages have also been reported to secrete IFN-γ upon stimulation via IL-12 and IL-18 (47) or via CD40 (48), which might further enhance M1 polarization through auto- or paracrine activity. Not all pathogens stimulate macrophages to switch from M2/heal to M1/inhibit, and some seem to suppress such a switch. In this circumstance, M2-type macrophages can stimulate T cells to make very different cytokines (such as IL-4, IL-13, and TGF-β) that cause B cells to become antibody-producing plasma cells (7, 29). These same cytokines also inhibit the M2 to M1 switch, and thus can amplify M2/heal activity (1). Of course, because there are many different pathogens invading different locales of hosts, there are always mixtures of M1/Th1 or M2/Th2-type responses as disease regression or progression occurs. In this connection, it is now known that tissue macrophages can arise from local renewal or from the blood (1, 4). The ontogeny of M1 and M2-type macrophages is not yet clear, and is beyond the scope of this article. Recent advances in metabolomics, defining resident microbiota, other areas, are opening up new horizons for understanding the myriad signals that regulate “immunity” (43). Though more is to be known, the aforementioned results have established important biochemical and physiologic elements that influence how macrophages serve to protect (or fail to protect) against infectious or other threats to host homeostasis.

## *In vitro* Versus *In vivo* Macrophage Conundrum

In addition to the basic macrophage functions necessary for life (such as SHIP), some investigators (primarily working *in vitro*) have employed ever-expanding lists of “markers” for macrophage “activation.” These include: cell surface antigens; expressed gene products; and other factors, and have created the notion that there are many different “varieties” of macrophages such as “M2 a, b, c,” “regulatory,” and “alternatively activated” macrophages (49–53). Unlike classifying macrophages by functions (e.g., M1/inhibit or M2/heal), the use of markers has created subsets without clear functional roles *in vivo*. Likewise, defining macrophage populations based on cytokine production patterns has caveats that are often overlooked. For example, macrophage cytokines such as IL-6 are “inflammatory,” yet they can be found in almost any site where macrophages are present (1). Indeed, the very presence of macrophages is inflammatory that raises questions about what “anti-inflammatory” macrophages are (47–49). Efforts to define macrophage “subsets” based on which cytokine (or agonist) has been used to stimulate them *in vitro* (such as IL-4 or IFN-γ) also leads to confusion since macrophages do not encounter isolated cytokines *in vivo*. Rather, they are constantly receiving hundreds of signals, the integration of which ultimately defines a cell’s behavior. Furthermore, because a selected cytokine can elicit a given macrophage reaction *in vitro* does not mean it has the same effect *in vivo*. For example, adding IL-4 to macrophages *in vitro* does increase M2-type activity (50). And IL-4 from T cells or innate cells can upregulate M2-type antibody responses (7, 29): what has been has been termed “alternative activation”). However, it is hard to ascribe M2-type responses in circumstances such as sterile wounds or tumors to “alternative activation” because little or no IL-4 is present (54, 55). Using T cell-derived cytokines to stimulate macrophages *in vitro* has also propagated the long-held notion that T cells are necessary to “activate” macrophages (23, 24). This perception runs counter to the observations that macrophages initiate and direct innate or adaptive responses (1). Another potential artifact of *in vitro* cultures is that macrophages can exhaust critical media components, and thus behave in ways (including dying) that are not observed *in vivo* where nutrients/other products are replenished (24).

Finally, the source of the “macrophages” being studied *in vitro* varies and has created confusion. Specifically, people studying humans have primarily used monocytes from blood because of convenience. And doing so has caused some to conclude there are major species differences in “macrophages,” including that humans seem less able (or unable) to produce iNOS/NO or arginase/ornithine (3, 56, 57). However, comparing monocyte-derived macrophages to tissue macrophages is an apples and oranges-type comparison. When human tissue macrophages have been examined, they do not appear fundamentally different from those of other vertebrate species (58).

Thus, a variety of pitfalls can make it difficult to translate results from *in vitro* cultures to understanding how macrophages function *in vivo*. In turn, rather than relying on “markers” or selected culture stimuli to try and define different macrophage “activation” states (59), it seems prudent to focus on characterizing macrophages by their known *in vivo* functions, such as SHIP (1).

## SHIP Functions to Navigate a Sea of Plasticity

Macrophage SHIP functions are associated with major differences in their metabolism and physiology (1). And hence, at the population level, macrophages must display considerable heterogeneity. “Plasticity” usefully describes the unique adaptability of macrophages as they change from, for example, producing a growth-promoting molecule (ornithine) to producing a growth-inhibiting molecule (NO) (12, 60). However, for some the concept of plasticity has morphed into a notion that macrophages are a fluid cell type that are always *only* changing (47–51). Like they say, “change is good” (humor intended). But, like changing clothes, it is not the changing that matters: it is the result. Perhaps, the clothes help one get a job, or, get a date, etc. And so it is with macrophages. As macrophages make major switches in their metabolism, they are “changing.” But, the changes in functional properties of macrophages can create illusory heterogeneity as illustrated in Figure 1B. Specifically, if a population of resting/resident macrophages (or a single macrophage, left) receives appropriate signals (e.g., LPS and/or IFN-γ) and commits to switching from M2/heal to M1/inhibit dominant activity, it takes the cell(s) several hours to accomplish this major change in metabolism. In turn, at any given time there will be a variety of different macrophages expressing different M2 and (increasingly in this example) M1-type activity. In turn, if one examines macrophages (or a single macrophage) at any given time there will be intermediate phenotypes in terms of marker or cytokine expression. Also often overlooked is that M1-type macrophages produce non-specific killer molecules (like NO) that inhibit or kill macrophages too (24). In turn, analysis of whole populations can create the additional illusion that M1-type have converted back to M2-type, when actually, they are dead/missing (1) In turn, examining macrophage populations (particularly *in vitro*) can create impressions of reversible plasticity or heterogeneity, but which are not based on what functions the macrophages have (e.g., M1/inhibit or M2/heal) (49). Thus, heterogeneity (or plasticity) is a means to an end. The “end” immunologists should strive for is identifying macrophages by their health-impacting functions (1).

## Summary

Immunology has and will continue to cure important diseases. And, the ability to culture macrophages *in vitro*, the expanding power of “transcriptome” analysis to examine thousands of genes, the capability of analyzing single macrophages, and other new technologies are providing necessary new information about the cellular biochemistry and physiology of leukocytes. But, as demonstrated here with macrophages, overemphasis on ambiguous “markers,” or analyzing whole populations of macrophages that are changing their functions, can create an illusion – a “sea of plasticity.” Therefore, to navigate this sea, it is critical to focus on SHIP functions (e.g., sample, heal, inhibit, and present) that importantly affect health. Doing so will help unleash the tremendous potential for usefully modulating innate immunity/macrophages against a variety of conditions ranging from cancer to atherosclerosis. To cure disease.

## Conflict of Interest Statement

The authors declare that the research was conducted in the absence of any commercial or financial relationships that could be construed as a potential conflict of interest.

## Footnotes

^1^Both macrophages and dendritic cells can direct the type of T cell response. So, the word “macrophage” will be used here to refer to both. Readers are directed elsewhere for discussions of macrophages and dendritic cells.
